# The Application of [^68^Ga]-Labeled FAPI-04 PET/CT for Targeting and Early Detection of Pancreatic Carcinoma in Patient-Derived Orthotopic Xenograft Models

**DOI:** 10.1155/2022/6596702

**Published:** 2022-08-05

**Authors:** He Zhang, Jiaze An, Pengpeng Wu, Caiqin Zhang, Yong Zhao, Dengxu Tan, Changhong Shi, Xu Ge

**Affiliations:** ^1^Division of Cancer Biology, Laboratory Animal Center, Fourth Military Medical University, Xi'an 710032, Shanxi, China; ^2^Training Center of Clinical Skills and Medical Staff, General Hospital of Northern Theater Command, Shenyang 110016, China; ^3^Department of Hepatobiliary and Pancreaticosplenic Surgery, Xijing Hospital, The Fourth Military Medical University, Xi'an 710069, China

## Abstract

[^18^F]FDG as a probe of PET/CT is a radiolabeled glucose analogue taken up by most cells, but its batch activity is limited. [^68^Ga]FAPI-04 is a promising alternative based on a fibroblast activation protein-specific inhibitor (FAPI) labeled with radiotracer FAP. Here, a series of databases suggested that FAP expression was significantly different in pancreatic cancer compared to normal tissue. The FAP-positive fibroblasts were evaluated around the tumor cells and the stroma. A patient-derived orthotopic xenograft (PDOX) model of pancreatic adenocarcinoma (PDAC) exhibits significantly higher quantitative uptake of [^68^Ga]FAPI-04 (*P* < 0.05) than [^18^F]FDG PET/CT in various organs. Because of relatively high (T/M) ratios, the [68Ga]FAPI-04 is excellent for B-mode ultrasound, NIRF, and PET/CT. Thus, [^68^Ga]FAPI-04 PET displayed a better tumor specificity and can be a potential application for the early detection of pancreatic cancer.

## 1. Introduction

Positron emission tomography/computed tomography (PET/CT) is an accurate and noninvasive tool that can detect pathophysiological changes early, especially in oncology. Changes occur before clinical symptoms and anatomical changes are detected by radiological techniques [1]. Currently, PET/CT may provide functional and morphological information for early oncological diagnosis [2]. Fluorine-18 fluorodeoxyglucose ([^18^F]FDG) as a probe of PET/CT is a radiolabeled glucose analogue taken up by most cells. It takes advantage of the propensity that most tumor cells consume high levels of glucose [3]. However, the accumulation of [^18^F]FDG by nonmalignant cells limits the detection of cancer in tissue with high native glucose uptake, and not all cancer cells preferentially metabolize glucose [4]. [^18^F]FDG lacks oncological specificity as activated immune cells in tumors or inflammatory lesions also accumulate the radiotracer [5].

Fibroblast activation protein (FAP) is highly expressed on cancer-associated fibroblasts in the tumor stroma and at very low expressed levels throughout the body [6]. Especially breast, colon, and pancreatic carcinomas are characterized by a strong desmoplastic reaction, which means almost the gross tumor mass can consist of stromal but not tumor cells [7]. Fibroblasts are present ubiquitously in the whole body but no or only a very low FAP expression. In contrast, cancer-associated fibroblasts are specifically characterized by the expression of FAP [8]. FAP inhibitors (FAPIs) have already been developed as targeted cancer drugs. Several FAPI molecules have been developed as targeted drugs against various types of cancer of epithelial origin after being radiolabeled with a radioactive atom. ^68^Ga-labeled FAPI-04 based on a FAP-specific inhibitor labeled with radiotracer was designed and may be a promising alternative molecule probe [9, 10].

Pancreatic ductal adenocarcinoma (PDAC) is a gastrointestinal lethal disease with high malignancy and poor prognosis, accounting for the fourth leading cause of cancer-related deaths worldwide, according to the American Cancer Society (ACS) [11]. It is the seventh most common malignant tumor in the gastrointestinal tract, with the incidence rate rising four times in the last two decades in China [12]. Patients with precancerous lesions and early PDAC typically exhibit no obvious symptoms. Accordingly, due to a lack of appropriate methods of early detection, it is not definitely diagnosed until the disease has progressed into advanced stages [13]. Only 20% of patients are diagnosed at an operative stage, contributing to limited therapeutic options. The poor 5-year survival rate is still below 8%, as reported in [14]. These poor survival rates have not changed significantly in nearly 40 years.

Biomarkers are efficient and crucial for the early detection of PDAC. Unfortunately, there are no validated biomarkers that are currently clinically available [15]. Medical imaging plays an essential role in the diagnosis of PDAC by allowing a comprehensive evaluation of the morphological, metabolic, and biological changes in the parenchyma and duct of the pancreas. Various imaging modalities may identify pancreatic cancer early [16]. Here, we established patient-derived orthotopic xenograft (PDOX) models of PDAC with early stage, which successfully mimic the symptomatology, pathology, and microenvironment of the tumor [17–19]. Then, comparisons between the current standard [^18^F]FDG PET and new developed [^68^Ga]FAPI-04 PET were approximated about radiation exposure of serial PET, also near-infrared fluorescence (NIRF) imaging and B-mode ultrasound were implied, in order to test the efficacy of the early detection of [^68^Ga]FAPI-04 probe. All these results demonstrated that [^68^Ga]FAPI-04 PET has better tumor specificity and can be a potential application for the early detection of PDAC.

## 2. Materials and Methods

### 2.1. Animals

Male, aged 6–8 weeks, BALB/c nude mice (purchased from Changzhou Kavins Experimental Animal Co. LTD, China) were used in this research. Nude mice were bred in an SPF-level barrier environment at the Laboratory Animal Center within the Fourth Military Medical University (FMMU) and acclimatized for up to 1 week prior to the commencement of orthotopic implantation. All efforts were made to minimize animal suffering, to reduce the number of animals used, and to utilize alternatives to *in vivo* techniques, if available. All procedures in this study were approved by the Laboratory Animal Welfare and Ethics Committee (No. 20013).

### 2.2. Clinical Specimens

All clinical pancreatic PDAC specimens were obtained from the Department of Hepatobiliary and Pancreaticosplenic Surgery at Xijing Hospital. The histology subtypes were poorly differentiated pancreatic carcinomas diagnosed as stage 1 and stage 2 PDAC respectively by two experienced doctors in the pathology department. The use of human tissue specimens in research was approved by the institutional review board (IRB) of the FMMU.

### 2.3. Preparation of Orthotopic Xenograft Models

To establish PDOX models, fresh patient specimens with PDAC were transplanted orthotopically into nude mice according to a published protocol in the first place [20]. When gross volume (*V*) was 400–600 mm^3^ as indicated in the following formula *V*=1/2 × (*w*^2^ × *l*)*w*^2^, tumors were harvested and cut into 1-mm [3] tissue block. The mice were anesthetized with 2% isoflurane. Routine disinfection was performed in a left lateral decubitus position. The block mixed with Matrigel was transplanted into a pancreatic vascular bed by surgical means to establish orthotopic xenograft models of PDAC.

### 2.4. PET/CT Imaging

Tumor-bearing mice were divided into two groups (*n* = 3/group). Mice were fasted for 48 h before imaging. Mice in group A were injected intravenously with 0.2 mCi [^18^F]FDG, whereas mice in group B were injected intravenously with 3.7–7.4 MBq of ^68^Ga-labeled FAPI-04. PET/CT static scans were performed at 15, 30, 60, 120, and 180 min with a nano-PET/CT system (Mediso, USA). All PET/CT images were processed and analyzed using Nucline nanoScan software (Mediso, USA). Radioactivity uptake per gram of tissue as a percentage of total injected radioactivity count (%ID/g) was calculated by using a three-dimensional volumetric region of interest with a threshold of 40%. The threshold was determined experimentally on images, reconstructed identically to the scans. PET/CT imaging was performed in the Department of Nuclear Medicine in Xijing hospital.

### 2.5. NIRF and B-Mode Ultrasound

Anesthetized tumor-bearing mice were subjected to B-mode ultrasound to detect tumor growth using VINNO 6 LAB System (Feiyinuo Technology (Suzhou) Co., LTD, China). From the image of ultrasound, the size of the tumor could be measured accurately. The volume was calculated as the formula mentioned above.

Heptamethine cyanine compounds NIRG IR-783 were provided by Dr. Leland W. K. Chung (Cedars-Sinai Medical Center, Los Angeles, CA, USA) [21]. Whole-body or organ-specific optical imaging was performed after the injection of IR-783(200 *μ*mol/mice), using the Caliper Lumina II Small animal optical imaging system (PerkinElmer, Waltham, MA, USA) equipped with NIRF filter sets (excitation/emission, 783/840 nm) [22].

### 2.6. Histology and Immunohistochemistry

Mice were euthanized by CO_2_ asphyxiation after NIRF imaging. Complete necropsy of the animals was performed, and tissues were fixed in 10% neutral buffered formalin (NBF) solution, embedded in paraffin, and sectioned at 3–5 *μ*m. Hematoxylin and eosin (H&E) staining was performed. Immunohistochemical analysis was performed using a 3-3′-Diaminobenzidine Development Kit (CWBio, Beijing, China) according to the manufacturer's instructions. The anti-human FAP-*α* antibody (ab207178, Abcam, Cambridge, UK) was diluted 1 : 250.

### 2.7. Statistical Analysis

Statistical analysis included the two-tailed Student *t*-test or one-way ANOVA with multiple comparisons and Bonferroni's post hoc test using GraphPad Prism v8.0 (GraphPad Software, USA). Data were represented as mean ± standard deviation (SD). The comparison of [^18^F]FDG PET/CT and [^68^Ga]FAPI-04 in various organs was done using paired Student *t*-test, and the tumor volume or ROI in each mouse between the two groups was evaluated using one-way ANOVA. Statistical significance was defined where the *P* value was less than 0.05.

## 3. Results

### 3.1. FAP Expression on Pan-Cancers

**Figure 1:**
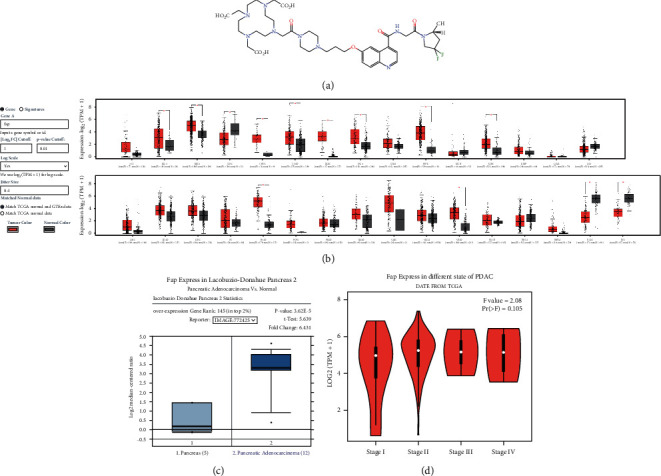
Characteristic of [^68^Ga]FAPI-04, the quinoline-based FAP-specific inhibitor. (a) Molecular formula of [^68^Ga]FAPI-04; (b) comparison of FAP expression between Pan-cancer and normal tissue. Note: data from TCGA and GTEx; (c) comparison of FAP expression between PDAC and normal tissue; and (d) FAP Expression in different stages of PDAC.

[^68^Ga]FAPI-04 is a new PET probe and quinoline-based FAP-specific inhibitor [23, 24]. The strategy of compound synthesis is described in detail in Figure S1, and the molecular formula is shown in Figure 1(a). FAP is a widely distributed antigen in epithelial tumor cells, expressed in 90% of epithelial tumor tissues and the microenvironment of several tumor types [25, 26]. Compared with normal, the expressions of FAP have significant differences on 13 of 31 Pan-cancers, such as breast, colon, and especially pancreatic carcinomas (Figure 1(b)). Based on TCGA and GTEx data, FAP expresses significantly different between PDAC and its normal (Figure 1(c)), but not different among the four stages of PDAC (Figure 1(d)). Together, these findings provide a rationale for the new probe based on FAP targeting the early stage of PDAC.

### 3.2. PET Imaging and Biodistribution

**Figure 2:**
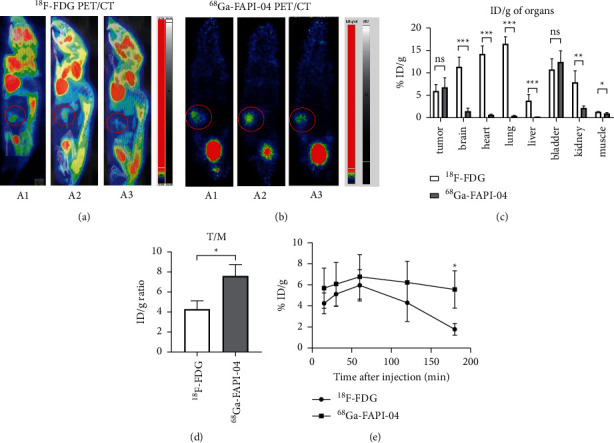
PET-based biodistribution analysis of comparing [^18^F]FDG PET and [^68^Ga]FAPI PET. (a) [^18^F]FDG PET/CT imaging; (b) [^68^Ga]FAPI-04 PET/CT imaging; (c) the ID/g of [^18^F]FDG and [^68^Ga]FAPI-04 in different organs 60 min after injection; (d) the ID/g ratio of tumor vs muscle. (e) The ID/g of [^18^F]FDG and [^68^Ga]FAPI-04 at different imaging time points (10 min, 20 min, 40 min, 60 min, and 180 min after injection) in mice with xenograft tumor. ^*∗*^*P* < 0.05, ^*∗∗*^*P* < 0.01, ^*∗∗∗*^*P* < 0.001, NS = not significant.

The new probe, [^68^Ga]FAPI-04, could have a relatively high-specific expression targeting tumor-associated fibroblast. In PET/CT imaging, the signal of orthotopic human PDAC carcinoma was both significant, no matter what probe was, as shown in Figures 2(a) and 2(b) marked in red. The tumors' location was just as expected. But in the [^68^Ga]FAPI-04 group, only the bladder had stronger tracer signals than the tumor's, not observed in the [^18^F]FDG group. In the organs of the brain, heart, lungs, kidney, and bladder, the signals of [^18^F]FDG were more significant than the tumor's (*P* < 0.05). Because of the high glycometabolism-related levels of organs, they had strong tracer signals. The tumors showed the tracer signal as well.

PET-based biodistribution was analyzed intraindividually by comparing [^18^F]FDG and [^68^Ga]FAPI-04 (Table 1). The quantitative tumor uptake of [^68^Ga]FAPI-04 PET was similar to that of the current oncologic [^18^F]FDG PET standard of reference (average %ID/g, 5.61 for [^18^F]FDG and 7.63 for [^68^Ga]FAPI-04, not statistically significant). In the organs of the brain (11.33 vs. 1.33, *P* < 0.001), heart (14.17 vs. 0.63, *P* < 0.001), lungs (16.33 vs. 0.40, *P* < 0.001), liver (3.85 vs. 0.13, *P* < 0.001), kidney (7.89 vs. 2.13, *P* < 0.01), and muscle (3.34 vs. 0.87, *P* < 0.05), the quantitative uptake was significantly different between the two groups. In contrast, the average ID/g presented no relevant differences in the quantitative uptake of the bladder (*P* > 0.05), as shown in Figure 2(c). Moreover, we analyzed the dynamic PET imaging indices, including the max of standardized uptake value, the tumor to muscle (*T*/*M*) ratios, and change in ID/g during PET lasting. The [^68^Ga]FAPI-04 range of *T*/*M* ratios for uptake was 6.43–8.66, and the [^18^F]FDG range was 3.42–5.08. The difference between the two groups was significant (*P* < 0.05), indicating a more effective cancer-specific uptake of the [^68^Ga]FAPI-04 probe captured by PET (Figure 2(d)).

The mice of each group examined 15 min to 180 min after injection demonstrated that both tracers reached their stable physiologic biodistribution at about 60 min. Tumor uptake declined about 75% from 60 min to 180 min after injection using [^18^F]FDG. Longer tumor retention was observed with [^68^Ga]FAPI-04 at 180 min (*P* < 0.05), as shown in Figure 2(e). Moreover, [^68^Ga]FAPI-04 tracers performed lower tumor-to-background ratios at 60 min after injection (data not shown).

### 3.3. Imaging of B-Mode Ultrasound and NIRF in Nude Mice with Orthotopic PDAC Xenograft

**Figure 3:**
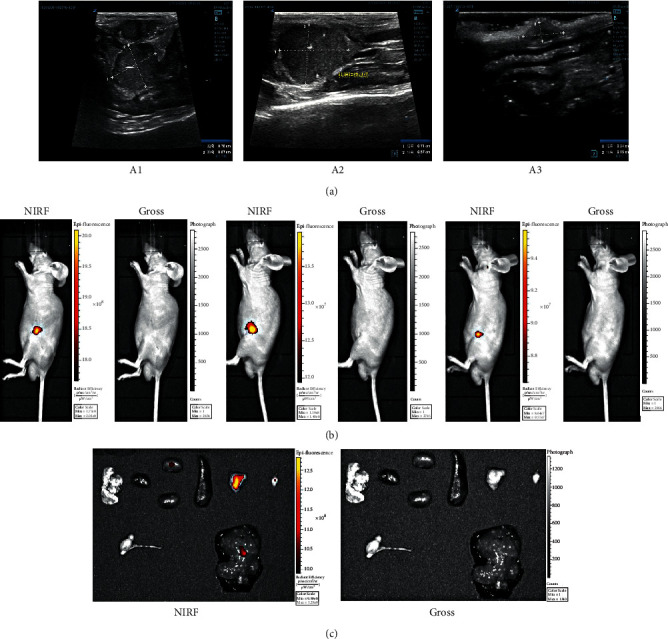
Imaging and targeting pancreatic orthotopic tumor of group A. (a) B-mode ultrasound imaging of tumors; (b) NIRF imaging of tumors; and (c) NIRF imaging of organs. The lungs, heart, kidney, spleen, tumor *in situ*, and inguinal lymph node are from left to right in the first row. The testes and spermaduct, and liver are in the second row respectively.

B-mode ultrasound has been an imaging technique commonly used in clinical practice for tumor preliminary monitoring. Most organs and their internal structure of mice were distinguished precisely in ultrasound images. So was the size and shape of the tumor range (Figure 3(a) and Figure S2). According to participants' judgments, B-mode ultrasound imaging was not tumor specific. For this, B-mode ultrasound was considered as a complementary method in clinical practice [13].

NIRF imaging is a completely promising tool for tumor imaging detected by targeting IR-783 dye, which could be readily detected within deep tissues by imaging modalities and it is effective for the detection of tumor location. They have shown great potential for both experimental and clinical imaging, including real-time visualization of certain anatomical structures during surgery [27]. The status of tumor location and metastasis properties could be preliminarily detected, according to the intensity and proportion of fluorescence signal (Figure 3(b) and Figure S2).

For further demonstration of the tumor's precise location and metastasis properties, most organs in abdominal cavity and thorax were dissected and separated from sacrificed mice (Figure 3(c) and Figure S2). Evidence for tumor cell recognition signal to pancreas showed high-affinity binding proteins with tumor-targeting properties. But, small amounts of fluorescent signal were also detected in the liver and kidney in some mice. Perhaps, a low concentration of NIRF locally remained in these abundant blood supply organs. No detectable activity was found in other organs. Evidence demonstrated the tumor growth at the predicted site precisely and without metastasis. NIRF imaging showed a significant signal of the pancreatic orthotopic tumor.

### 3.4. Histological and Immunohistochemical Analysis

**Figure 4:**
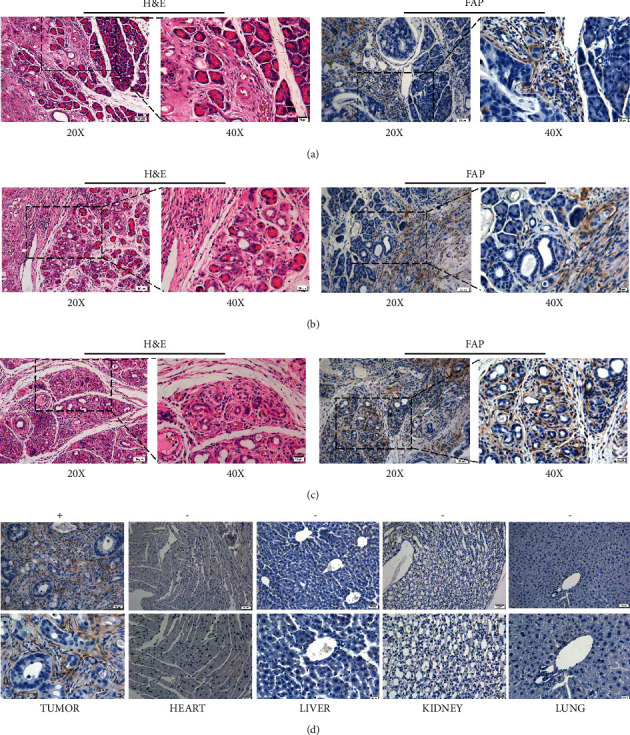
Histological and immunohistochemical of PDOX models. (a) Patient-derived tumor tissue was partly invading mouse's pancreas islet. The border and the construction of islet was still clear. (b) Tumor tissue had totally invaded pancreas islet. The border and the construction of islet was hazy. (c) Tumor cells had totally occupied pancreas islet. The whole islet was full of tumor cells. (d) Antigen FAP was positive in the fibroblasts of tumor stroma, but negative in the heart, liver, kidney and lungs.

PDAC is the most common type of pancreatic malignancies, accounting for about 80–90% of all pancreatic cancers, which contains ductal structures per gland of differentiation degree, abundant stromal cells responsible for fibrotic stroma, and mucoid epithelium. The pancreatic tissues of normal mice display a clear and normal structure of the islets of Langerhans, which contained abundant and full form islets. Fortunately, H&E staining of our models confirmed all the three different phases and classified structure of tumor and pancreatic islet. In the first phase, part of neoplasms extended into the peripancreatic soft tissue. The boundary of two tissues were still clear to distinguish (Figure 4(a)). In the second phase, pancreatic tissue was partly encapsulated (Figure 4(b)). The boundary of two tissues were not clear to distinguish. But the pancreatic tissues were presented as normal structure with little histopathological changes. In the last phase, pancreatic tissue was indistinctly demarcated from tumor tissue. The border of the two different tissues was inconspicuous. The pancreatic islet's structure was completely disrupted by tumor (Figure 4(c)).

Antigen FAP was mentioned for cancer-associated fibroblasts in the tumor stroma [28]. The positive fibroblasts were obviously found around the tumor cells and just in the stroma. There were negative fibroblasts in other organs like heart, liver, kidney, and lungs (Figure 4(d)). Antigen FAP has already been developed as targeting cancer tracer, and it could show different xenograft tumor invasion into islets. These indicated that [^68^Ga]FAPI-04 tracer possessed the ability of tumor specificity with fibroblasts in the stroma [29].

### 3.5. Sensitivity and Tumor Specificity of Three Type of Imaging Methods

Three type of imaging methods were compared by tumor' status and technical requirements (Table 2). The analysis indicated ultrasound was better method for monitoring tumor growth just at orthotopic model in mice, because of the convenience in tumor measurement and equipment mobility. But there was no specificity of tumor or metastasis 30. It is the Achilles heel of this method for qualitative evaluation of PDAC. NIRF overcame shortcomings of ultrasound method. It had sensitivity and specificity of tumor, which is also suitable to metastasis of tumor. But they were not accurate and convenient for the early detection of screening, because of the invasive procedure and fluorescent marker reaction. [^68^Ga]FAPI-04 PET/CT, compared with [^18^F]FDG, had a better signal-to-noise ratio (SNR) and a higher *T*/*M* ratio. Meanwhile, PET/CT could continuously and precisely measure tumors, especially for sensitivity and tumor specificity. It was the most comprehensive method for the early detection of PDAC, not only at monitoring the size and shape of tumors, but also at detecting tumor specificity and metastasis.

## 4. Discussion

Pancreatic carcinoma is a lethal disease with a poor and dismal prognosis, which might be caused by complex biology and tumor heterogeneity. Difficulties are also directly related to the lack of early detection and optional treatment [2, 30]. Not like other cancers, PDAC is short of validated biomarkers that are currently clinically available, including auxiliary diagnostic index CA19-9 [11, 16, 31]. The abovementioned imaging diagnosis has been widely used in preclinical or clinical management of pancreatic cancer [32]. Specially, PET/CT and PET/MR have showed excellent performances for the initial staging, treatment determination, therapy response, and prognosis evaluation of pancreatic cancer and contributed to improve the tumor outcome [1]. [^18^F]FDG, a glucose metabolism probe, is the clinical routine for diagnosis [5]. However, it still remains several drawbacks in pancreatic cancer early diagnosis. Both of the chronic and acute pancreatitis have high uptake of radionuclide tracer. That might lead to false-positive interpretations [33]. Another reason that might have some effect on the application of [^18^F]FDG PET is tricky diagnosis on hyperglycemic patients. Because, the uptake of the tracer is suppressed as high serum glucose competitive inhibition. Therefore, the exploitation of new molecular probes, which could target pancreatic cancer specifically and overcome the limitations of [^18^F]FDG, are urgently needed and should have great clinical significance for early detection.

The tumor stroma of pancreatic cancer comprised of cancer-associated fibroblasts (CAFs), inflammatory cells, blood vessels, and extracellular matrix (ECM). All abovementioned could occupy up 90% of the entire tumor mass [12]. PDAC is characterized by the existence of an ample desmoplastic stroma. There is a general consensus that CAFs are crucial for the formation of the desmoplastic stroma; these cells promote tumor progression and metastasis and might also be implicated in chemoresistance and immunosuppression [34]. The results of bioinformatic analysis implied that FAP expresses significantly different between PDAC and normal tissue, but not different among the four stages of PDAC. The new probe, FAPI-04, evolved from fibroblast activation protein inhibitor has already been developed as a promising new multi-tumor target for diagnostics. Though the present studies of [^68^Ga]FAPI biodistribution demonstrated a high uptake in primary PDAC, lymph nodes, and distant metastases, with low activity in healthy tissues, there was still some negative PDAC detection at the early stage. There is a lot of potential to allow for further exploration of initial stage diagnosis and treatment strategies before clinical and preclinical applications, especially to establish a more preclinical model of early PDAC to test the efficacy of the [^68^Ga]FAPI probe.

Herein, in this study, PDOX models derived from patients diagnosed as early (stage 1 and stage 2) PDAC were established successfully. This model mimicking simulated the *in situ* tumor growth and maintained the consistency of microenvironment of the primary tumors, established through the con-injection of cancer cells and PSCs, which is more consistent with the clinical pathological characteristics of human pancreatic cancer. Meanwhile, it also possessed the heterogeneity characteristics inside tumors. Different from convenience of evaluating subcutaneous bearing tumors in PDOX models, PDOX model tumors usually grew *in vivo* and were lack of corresponding methods to evaluate the process of tumor initiation, development, and metastasis [35].

Furthermore, the results of immunohistochemical staining revealed abundant FAP expression in the stroma of xenografts model, compared with the negative expression of other organs. Thus, we tested the targeting and specificity of the probe on the PDOX model of PDAC in early stage. Compared with clinic commonly used [^18^F]FDG, results revealed that there was no significant difference in tumor specificity, but there were lower signal-to-noise ratio and higher effective uptake of tumor after injection. Consequently, we were the pioneers to evaluate PDOX models of PDAC by using [^68^Ga]FAPI-04 PET/CT and make the comparison with the current standard [^18^F]FDG [36]. Some researchers have reported that [^18^F]FDG PET/CT is able to influence clinical decision, but unfortunately with a low specificity of 76% for the detection of PDAC [10]. [^68^Ga]FAPI-04 is designed and developed as a well-recognized tracer targeting for FAP [37, 38]. A few human cases demonstrated [^68^Ga]FAPI-04 high-contrast tumor imaging and possible appropriateness as a pan-tumor agent [39]. Moreover, comparison of uptake value in major organs verified the new probe had better tumor specificity and higher *T*/*M* ratio. The glycometabolism of tumor cells is much higher than normal tissues. They need to increase the glucose uptake to satisfy the metabolism demand. So the glucose analogue [^18^F]FDG molecular tracer could enrich on tumor cells specifically in ideal situation [5]. From the results, [^18^F]FDG PET does highlight the solid tumors. However, some obvious red regions in other normal organs had more stronger uptake signal than the tumor's region, which indicated that glycometabolism of these normal tissues was more active than tumors. In quantitative icon, we could clearly figure out that the ID/g of the brain, heart, lungs, kidney, and bladder was significantly higher than the liver. Fortunately, the signal of tumor did not been covered by some normal organ in the abdominal cavity, which might explain why the early diagnosis by [^18^F]FDG PET/CT of pancreatic cancer is not satisfactory.

In addition, we used another two imaging technologies to compare with [^68^Ga]FAPI-04 PET/CT—NIRF optical imaging and B-mode ultrasound scan. According to our previous work, heptamethine carbocyanine (HC) dyes, a class of near-infrared fluorescent (NIRF) dyes, including IR-783, and MHI-148, are specifically recognized by various tumor cells, with tumor targeting and therapy functions [20]. Similarly, both NIRF and PET modalities exploit tumor-specific tracers (either labeled with a fluorescent protein or radioisotope), targeting biomarkers abundantly present on tumor tissue and absent on (or minimally expressed by) benign or inflamed tissue [40]. But the sensitivity of NIRF was highly associated with tumor location and depth, due to the wavelength coverage of NIRF. In our study, the NIRF probe detected the occurrence of pancreatic cancer, perhaps it is because mice are smaller size and have a shallower pancreas. Perhaps it is because mice are smaller size and have a shallower pancreas. As a noninvasive technology, B-mode ultrasound has been used in early diagnosis for many years. The upgraded B-mode ultrasound could evaluate tumor-bearing mice models [41] and efficacy with accurate imaging, in which organs and their internal structure could be distinguished and so was the size and shape of bearing tumors in the abdominal cavity. The advantages of ultrasound were conducted in real time and compatible with other imaging as a complementary modality to PDAC. In this study, although B-mode ultrasound could tell precise tumor size and location, it has no tumor specificity, which could provide multiple imaging methods for PDAC. It had permitted visualization of neoplasms, but without tumor specificity. In the context of early detection, imaging-based approaches can be grouped into traditional and nontraditional applications. Furthermore, particular emphasis on areas of unmet need of imaging technologies is discussed. A comprehensive comparison of the two imaging methods was made to further illustrate, whereas tumor-specific [^68^Ga]FAPI-04 PET/CT may contribute to improved surgical planning, stratification, and early diagnosis as well as therapy response monitoring after neoadjuvant treatment [29, 39].

Present studies of [^68^Ga]FAPI biodistribution demonstrated a high uptake in primary PDAC, lymph nodes, and distant metastases, with low activity in healthy tissues [39]. However, there were still some negative detection of PDAC at the early stage. It still needed more exploration of initial stage diagnosis and treatment strategies before clinical and preclinical applications [24], especially to establish more preclinical model of early PDAC to test the [^68^Ga]FAPI probe.

There are already some studies in humans with the same objective presented. For example, both Kaghazchi [42] and Deng et al. [33] reported one patient with advanced pancreatic adenocarcinoma, imaged with [^18^F]FDG and [^68^Ga]FAPI PET/CT scans, respectively. They found the intensity and SUVs of the lesions were higher in [^68^Ga]FAPI scan compared with [^18^F]FDG. [^68^Ga]FAPI showed higher contrast in lesion-to-background ratio and increased detection rate of metastatic sites. Those two cases highlight that [^68^Ga]FAPI PET/CT may be superior to [^18^F]FDG PET/CT for identifying primary and liver metastases lesions. However, those two studies belong to a single case report, and the relative results lack comparison of a large number of clinical samples.

This study focused on better understanding the role and efficacy of ^68^Ga-FAPI-04 PET/CT imaging in the early detection of pancreatic carcinoma in PDOX models. This model successfully mimic the symptomatology, pathology, and microenvironment of the tumor, to carry out systematic and standardized experimental research. Both the targeting and the detection efficiency of [^68^Ga]-labeled FAPI-04 PET/CT on early stage PDAC were concerned. Subsequently, we made comparisons between [^18^F]FDG and [^68^Ga]FAPI-04 by PET; in addition, other different imaging techniques such as near-infrared fluorescence imaging (NIRF) and B-mode ultrasound are implied, in order to confirm the efficacy of the early detection of [^68^Ga]FAPI-04 probe for PDAC model.

## 5. Conclusion

We successfully established patient-derived orthotopic xenograft (PDOX) models of PDAC with early stage, which successfully mimic the symptomatology, pathology, and microenvironment of the tumor. The uptake of [^68^Ga]FAPI-04 in these PDOX models was significantly increased compared to [^18^F]FDG PET/CT. Being relatively high (*T*/*M*), [^68^Ga]FAPI-04 is excellent for B-mode ultrasound, NIRF imaging, and [^18^F]FDG PET/CT.

## Figures and Tables

**Table 1:** Biodistribution analysis of comparing [^18^F]-FDG PET and [^68^Ga]FAPI PET.

	^18^F-FDG group			^68^Ga-FAPI-04 group		
	Mouse 1	Mouse 2	Mouse 3	Mouse 4	Mouse 5	Mouse 6
Tumor	5.74	7.51	4.58	8.82	4.58	6.86
Brain	9.13	11.34	13.45	0.93	2.19	1.32
Heart	12.45	16.06	14.01	0.51	0.62	0.83
Lungs	16.32	14.92	18.06	0.47	0.31	0.52
Liver	2.45	3.89	5.12	0.14	0.21	0.16
Bladder	10.12	8.66	13.34	14.18	13.48	9.56
Kidney	10.41	5.27	7.99	2.15	2.63	1.82
Muscle	1.41	1.32	1.24	1.2	0.92	0.71

**Table 2:** The comparison of sensitivity and tumor specificity of three type of imaging methods.

		B-mode ultrasound	PET/CT	NIRF
Tumor status	Location	+	+	+
	Size	+	+	−
	Shape	+	+	−
	Metastasis	−	+	+

Technical requirements	Invasive	−	+	+
	Substrates	−	+	+
	Reaction time of substrate	−	1 h	24 h
	Anesthesia	+	+	+
	Equipment mobility	+	−	−
	Professional operation	+	+	+
	Continuous monitoring	+	+	+
	Convenience	+	−	−

## Data Availability

The data that support the findings of this study are available from the corresponding author.

## Supplementary Materials

Supplementary Materials Fig S1. Strategy of [^68^Ga]FAPI-04 compound synthesis. Scheme 1 depicts the initial synthesis of FAPI-01 which was achieved by performing a Br/Li-exchange with n-butyllithium at 5-bromoquinolie-4-carboxylic acid (1) and quenching with elemental iodine to obtain iodoquinoline (2). This compound was coupled to the Gly-Pro-CN fragment by HBTU/HOBt activation to provide non-radioactive reference material of FAPI-01 (3). To enable radiolabeling by the incorporation of radiometals, the chelator DOTA was chemically linked to the basic scaffold of the FAP inhibitor. Fig S2. Imaging and targeting pancreatic orthotopic tumor of Group B. (A) B-mode ultrasound imaging of tumors; (B) NIRF imaging of tumors; (C) NIRF imaging of organs. From left to right, successively were heart, liver, spleen, lungs, kidney, testes and spermaduct, tumor *in situ*, and inguinal lymph node.
