# Supplementary material for: The Application of [68Ga]-Labeled FAPI-04 PET/CT for Targeting and Early Detection of Pancreatic Carcinoma in Patient-Derived Orthotopic Xenograft Models
Source: Contrast Media Mol Imaging. 2022 Aug 5;2022:6596702. doi: 10.1155/2022/6596702 (PMC9410842; doi:10.1155/2022/6596702)
Supplement: Supplementary Materials — Fig S1. Strategy of [68Ga]FAPI-04 compound synthesis. Scheme 1 depicts the initial synthesis of FAPI-01 which was achieved by performing a Br/Li-exchange with n-butyllithium at 5-bromoquinolie-4-carboxylic acid (1) and quenching with elemental iodine to obtain iodoquinoline (2). This compound was coupled to the Gly-Pro-CN fragment by HBTU/HOBt activation to provide non-radioactive reference material of FAPI-01 (3). To enable radiolabeling by the incorporation of radiometals, the chelator DOTA was chemically linked to the basic scaffold of the FAP inhibitor. Fig S2. Imaging and targeting pancreatic orthotopic tumor of Group B. (A) B-mode ultrasound imaging of tumors; (B) NIRF imaging of tumors; (C) NIRF imaging of organs. From left to right, successively were heart, liver, spleen, lungs, kidney, testes and spermaduct, tumor in situ, and inguinal lymph node. [file 6596702.f1.docx]

Supplementary


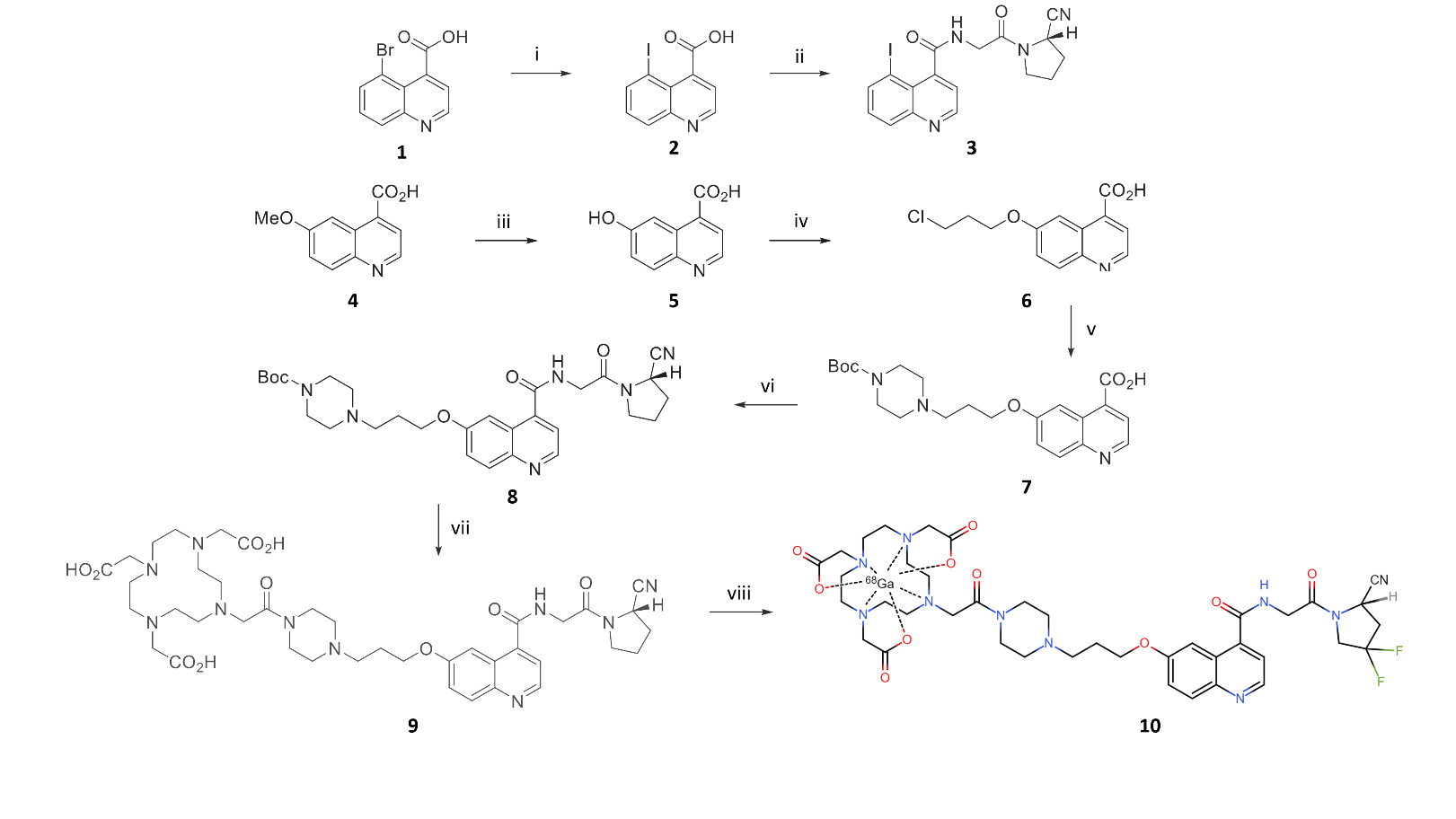


**Fig S1. Strategy of [^68^Ga]-FAPI-04 compound synthesis.**

Scheme 1 depicts the initial synthesis of FAPI-01 which was achieved by performing a Br/Li-exchange with n-butyllithium at 5-bromoquinolie-4-carboxylic acid (1) and quenching with elemental iodine to obtain iodoquinoline (2). This compound was coupled to the Gly-Pro-CN fragment by HBTU/HOBt activation to provide non-radioactive reference material of FAPI-01 (3).

To enable radiolabeling by incorporation of radiometals, the chelator DOTA was chemically linked to the basic scaffold of the FAP-inhibitor. Modifications at the 6-position of the quinoline-4-carboxylic acid are well tolerated without impairing target affinity and specificity. Therefore, a bifunctional linker was attached to the hydroxyl group of 5 via an ether linkage, leading way to the synthesis shown in Scheme 2. Ready available 1-bromo-3-chloropropane was chosen to create a spacer, which is unharmed during the saponification of the simultaneously formed ester bond at the end of the one-pot-process. Compound 6 was converted to the N-Boc protected quinolinecarboxylic acid 7 which was further coupled to H-Gly-Pro-CN by HBTU. Due to the high hygroscopicity of the free amine, compound 8 was directly converted to FAPI-02 (9) after the Bocremoval, solvent exchange and neutralization of excess p-toluenesulfonic acid. 2 hydrogen was replaced by 2 fluorine on the pentamethylene of 3, so called FAPI-04. ^68^Ga was chemically linked to FAPI-04 (10)

i) nBuLi, then I2, THF;

ii) HBTU/HOBt, DIPEA, HGly-Pro-CN, DMF

iii) aq. HBr 48%, 130 °C;

iv) 1-bromo-3-chloropropane, Cs2CO3, DMF then 6 M NaOH;

v) 1-Boc-piperazine, KI, DMF;

vi) HBTU/HOBt, DIPEA, H-Gly-Pro-CN, DMF;

vii) TosOH, MeCN, then DOTA-PNP, DIPEA, DMF;

viii) ^68^Ga

**
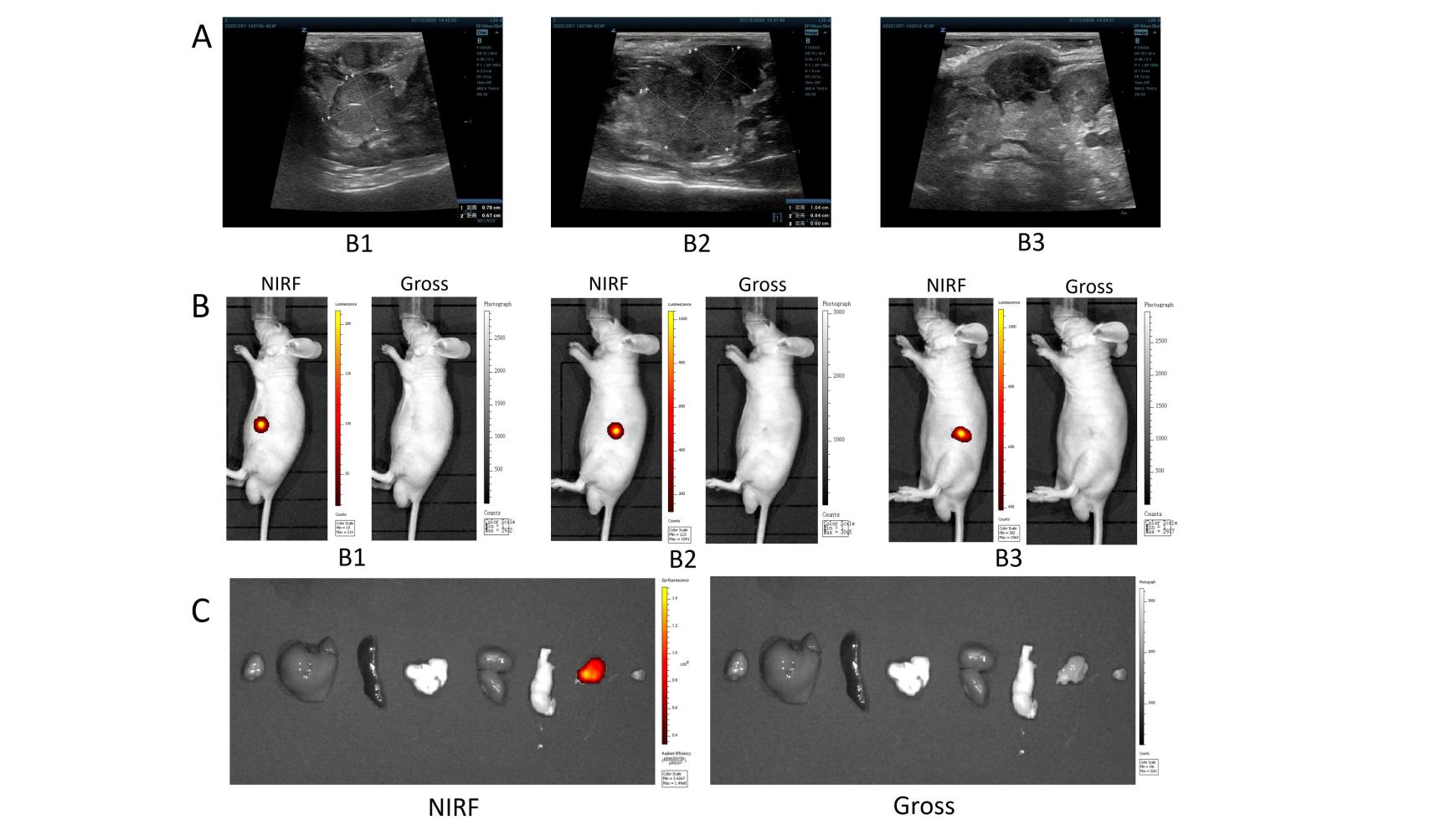
**

**Fig S2. Imaging and targeting pancreatic orthotopic tumor of Group B.**

(A) B-mode ultrasound imaging of tumors; (B) NIRF imaging of tumors; (C) NIRF imaging of organs. From left to right, successively were heart, liver, spleen, lung, kidneys, testes and spermaduct, tumor *in situ*, and inguinal lymph node.
